# Coordination of GPR40 and Ketogenesis Signaling by Medium Chain Fatty Acids Regulates Beta Cell Function

**DOI:** 10.3390/nu10040473

**Published:** 2018-04-12

**Authors:** Julien Benjamin Pujol, Nicolas Christinat, Yann Ratinaud, Claudia Savoia, Siobhan E. Mitchell, El Hadji M Dioum

**Affiliations:** 1 Islet Function, Nestlé Institute of Health Sciences (NIHS), EPFL Innovation Park, 1015 Lausanne, Switzerland; JulienBenjamin.Pujol@rd.nestle.com (J.B.P.); Claudia.Savoia@rd.nestle.com (C.S.); 2 Lipidomics, Nestlé Institute of Health Sciences (NIHS), EPFL Innovation Park, 1015 Lausanne, Switzerland; Nicolas.Christinat@rd.nestle.com; 3 Natural Bioactives Screening, Nestlé Institute of Health Sciences (NIHS), EPFL Innovation Park, 1015 Lausanne, Switzerland; Yann.Ratinaud@rd.nestle.com; 4 Brain Health, Nestlé Institute of Health Sciences (NIHS), EPFL Innovation Park, 1015 Lausanne, Switzerland; esiobhanmitchell@gmail.com

**Keywords:** insulin secretion, mitochondria, medium chain fatty acid, lipotoxicity, ketogenesis, BHB, GPR40

## Abstract

Diabetes prevalence increases with age, and β-cell dysfunction contributes to the incidence of the disease. Dietary lipids have been recognized as contributory factors in the development and progression of the disease. Unlike long chain triglycerides, medium chain triglycerides (MCT) increase fat burning in animal and human subjects as well as serum C-peptide in type 2 diabetes patients. We evaluated the beneficial effects of MCT on β-cells in vivo and in vitro. MCT improved glycemia in aged rats via β-cell function assessed by measuring insulin secretion and content. In β-cells, medium chain fatty acid (MCFA)-C10 activated fatty acid receptor 1 FFAR1/GPR40, while MCFA-C8 induced mitochondrial ketogenesis and the C8:C10 mixture improved β cell function. We showed that GPR40 signaling positively impacts ketone body production in β-cells, and chronic treatment with β-hydroxybutyrate (BHB) improves β-cell function. We also showed that BHB and MCFA help β-cells recover from lipotoxic stress by improving mitochondrial function and increasing the expression of genes involved in β-cell function and insulin biogenesis, such as Glut2, MafA, and NeuroD1 in primary human islets. MCFA offers a therapeutic advantage in the preservation of β-cell function as part of a preventative strategy against diabetes in at risk populations.

## 1. Introduction

The incidence and prevalence of Type 2 diabetes increases with age [1], and more than 30% of the elderly population in the United States are either diabetic or prediabetic [2]. Several factors contribute to the high prevalence of diabetes in the elderly population [3], including age-related changes in carbohydrate metabolism, reduced glucose-induced insulin release and β-cell dysfunction as well as peripheral insulin resistance due to increased adiposity and reduced physical activity [3,4]. Type 2 diabetes has been associated with insulin resistance induced by obesity and a sedentary lifestyle. Typically, most insulin-resistant subjects are able to increase their β-cell secretory capacity to meet the increased insulin demand and therefore, do not develop diabetes. However, failure of this β-cell compensation leads to type 2 diabetes [5,6]. The compensatory mechanism, characterized by increased β-cell replication and hyperplasia, declines with aging, making β-cell dysfunction a critical determinant of type 2 diabetes [7]. Several clinical studies have shown that the functional decline of β-cells with age results from a reduction in regeneration and adaptation that is needed to overcome age-associated insulin resistance [8,9], ultimately resulting in a reduced pancreatic β-cell mass and function [10]. Dietary intervention is a first approach to manage and/or prevent diabetes, and a reduction in carbohydrate intake or caloric restriction such as intermittent fasting [11] has been shown to improve β-cell function in obese and prediabetic rats [12]. These two interventions increase hepatic ketogenesis [13], and ketones bodies are known to improve cell survival and mitochondrial function in neurons and potentially in β-cells [14,15,16]. Medium-chain triglycerides are an important part of a ketogenic diet and have various health benefits [15,17]. In general, fatty acids are used a substrate for energy production, and catabolic breakdown by β oxidation yields acetyl-CoA, which can be used as a substrate for the carboxylic acid cycle and in oxidative phosphorylation to produce ATP, CO_2_ and H_2_O. In the liver in particular, during fasting or low carbohydrate availability, acetyl-CoA can be diverted to form ketone bodies (acetoacetate and β hydroxybutyrate) [13]. The medium chain fatty acids, octanoic acid (C8), and decanoic acid (C10), are found in the diet as medium chain triglycerides (MCT). After hydrolysis in the gut, the MCTs enter the bloodstream in the form of free fatty acids which are transported to the liver where most ketone bodies production takes place [18]. Unlike long-chain fatty acids, medium chain fatty acids (MCFA) are more water-soluble and are rapidly absorbed and oxidized [19] before being transported into mitochondria independently of carnitine [20]. Rats subjected to a MCFA-rich diet [16] show higher levels of circulating ketones and free fatty acids that have various effects on insulin secretion and glucose homeostasis [21,22]. Besides being an essential energy source, fatty acids function as signaling molecules that regulate various cellular processes based on the carbon length degree of saturation. Small chain fatty acids (C2–C6) are physiological ligands for fatty acid receptor (FFAR)2/GPR43 and FFAR3/GPR41 [23], whereas long chain fatty acids (C14 and up) signal through FFAR1/GPR40 and FFAR40/GPR120 [24]. Depending on the chain length, medium chain fatty acids have a low to medium range affinity for GPR40 [24], and activation of the receptor not only directly potentiates glucose stimulated insulin secretion in pancreatic β-cells, but also indirectly, through increased GLP1 and GIP1 secretion in the gut [24,25,26] which has beneficial effects on glucose homeostasis [27]. Dietary MCFAs enhance thermogenesis and fat oxidation, thus reducing fat deposition in animal and human subjects [28]. Activation of GPR40, coupled to the Gα_q_ subunit, triggers a signaling cascade that activates phospholipase C-β (PLCβ) followed by hydrolysis of phosphoinositol (PIP2)-containing membrane lipids to generate inositol 1,4,5-trisphosphate (IP3) and diacylglycerol (DAG). IP3, via the IP3 receptor in the ER, mediates the increase in intracellular Ca^2+^ levels that occurs after GPR40 activation [29]. Recently, MCFA have been shown to modulate mitochondrial enzyme function in neuroblastoma cell lines [30]. Considering the functional similarities between neurons and β-cells [31], we investigated the protective role of fatty acids and ketone body β-hydroxybutyrate (BHB) in β-cells. In the present study, aged rats, treated with MCT, composed of a 40:60 ratio of C8:C10, showed improved glycemia and β-cell function compared to the control rats fed with a sunflower oil-supplemented diet. Mechanistically, the MCFA C8:C10 supplementation coordinated C10-induced GPR40 activation and C8-induced mitochondrial ketogenesis in β-cells, improving function and increasing functional recovery from lipotoxic stress in human islets. Our data show that MCFAs have the therapeutic ability to preserve β-cell function in patients with a high prevalence to type 2 diabetes.

## 2. Materials and Methods

### 2.1. Cell and Islet Culture

Rodent β-cell line INS1E were maintained in DMEM and RPMI-1640 medium (Life Technologies, Thermo Fisher Scientific Inc., Waltham, MA, USA), respectively [32]. All cultures were kept in a humidified atmosphere at 37 °C and 5% CO_2_. Primary human islets were received from the Human Islets for Research consortium (HIR)^®^ distributed by Tebu-Bio SAS (Le Perray en Yvelines, France). The human islets were isolated from healthy donors that had been approved for research. Before the experiments, isolated islets were allowed to recover overnight at 37 °C and 5% CO_2_ in RPMI-1640 10% FCS, 2 mM Glutamax, 100 U/mL Penicillin, 100 µg/mL Streptomycin and 50 µM β-mercaptoethanol and treated accordingly with up to 500 μM MCFA.

### 2.2. RNA Isolation and qPCR

Total RNA was prepared with miniRNA Kit Plus (QIAGEN, Hilden, Germany). RT-PCR was performed with the Applied High Capacity cDNA Synthesis kit (Thermo Scientific Inc., Waltham, MA, USA), and cDNA was used for qPCR analysis. The target gene expression was evaluated using Power SYBR Green PCR Master Mix (Applied Biosystems, Foster City, CA, USA). PCR was carried on LightCycler 480 Real-Time PCR Systems (Roche Diagnostics, Mannheim, Germany) using a LightCycler 1536 SYBR green (Roche) [33]. Transcript levels were normalized to cyclophilin B. Relative fold change in expression was calculated using the ΔC_T_ method. For relative transcript quantification, each cDNA sample was run on a 4-point standard curve as to assure a PCR efficiency of ≥95%.

### 2.3. Immunocytochemistry and Confocal Microscopy

Pancreatic sections from treated, old rats were washed in PBS, fixed with 4% PFA overnight and then quenched with 0.125 M glycine. After washing, cells were permeabilized with 0.25% Triton X-100 and blocked with blocking buffer (5% Donkey Serum; 1% BSA; 0.05% Triton) for at least 30 min. Cell were incubated at 4 °C with goat anti-Insulin (Santa Cruz, 1/50). Secondary antibodies (Alexa Fluor, Molecular Probes) were diluted and incubated in blocking buffer for 1 h at RT. Nuclei were counterstained with DAPI (1/20000), and coverslips were mounted in Pro Diamond Mounting Media (Thermo Scientific Inc., Waltham, MA, USA) on microscopy slides. Images were acquired using a Leica SP8 confocal microscope.

### 2.4. IP1 Measurement Assay

Briefly, the activation of GPR40 receptors in stable Chem1-GPR40 cells induced diacylglycerol (DAG) and inositol trisphosphate (IP3) production. The rapid degradation of IP3 was inhibited by lithium chloride (LiCl) addition in the assay. Cellular IP1 content accumulation was measured using the competitive immunoassay based HTRF^®^ IPone-Gq kit (Homogeneous Time Resolved FRET from Cisbio, Codolet, France). IP1 produced by GPR40 activation competes with an IP1 analog coupled to a d2 fluorophore (acceptor) for binding to an anti-IP1 monoclonal antibody labeled with Europium Cryptate (donor). Upon excitation at 330 nm, the Europium Cryptate emits light at 620 nm. When the IP1 analog binds to the anti-IP1 monoclonal antibody the donor and acceptor come in close proximity, and fluorescence resonance energy transfer (FRET) occurs, leading to signal emission at 665 nm from the donor. This signal is inversely proportional to the amount of phosphorylated substrate. The ratio between 665/620 was measured using a Synergy neo multimode reader (BioTek, Winooski, VT, USA) and results are expressed as Ratio (665_signal_/620_signal_ x − 10,000). Chem1-GPR40 cells (10,000 per wells) were plated in a 384 Corning^®^ (#3657) in 50 μL culture medium for 24 h. Cells were then washed twice with 50 μL of assay buffer containing 146 mM NaCl, 10 mM HEPES, 5.5 mM glucose, 4.2 mM KCl, 1 mM CaCl_2_, 0.5 mM MgCl_2_ and 50 mM LiC,l and cells were then starved for 30 min in 20 mL of assay buffer before treatment. A volume of 10 µL of a 3× concentration of each of the tested compounds was then prepared in assay buffer (1% DMSO final) and added to the cells for 90 min. The reaction was stopped by the addition of 6 mL of IP1 d2 and 6 mL of anti-IP1 in lysis buffer. After 24 h, the plate content was transferred to a white, low volume, 384-well plate Corning^®^ (#3824). After an excitation at 330 nm, emission signals at 620 nm and 665 nm were recorded, and a negative ratio (665_signal_/620 _signal_ x − 10,000) was calculated.

### 2.5. Ketone Measurements

Analysis of 3-hydroxybutyric acid (BHB) was done with LC-MS, performed on an I-Class UPLC system (Waters Corp., Milford, MA, USA) coupled to a Thermo Scientific Q Exactive Plus mass spectrometer (Thermo Scientific, Bremen, Germany) operating in negative mode over the mass range 65–600 *m*/*z* at a resolving power of 35,000 (at *m*/*z* = 200), as previously described [15]. INS1E cells grown to confluence in 6-well plates were treated with the vehicle (DMSO), the GPR40 antagonist GW1100 (50 nM), the PLCβ inhibitor U73122 (2 μM), the IP3 receptor inhibitor (-) Xestospongin C (3 μM) or the l-type Ca^2+^ channel inhibitor Nifedipine (0.1 μM) for 15 min prior to incubation with 200 µM MCFA for 2 h in RPMI medium with 1 mM glucose. BHB was measured in the media by chromatographic separation performed on an Acquity UPLC BEH C8 column (1.7 mm, 100 × 2.1 mm; Waters Corporation, Milford, MA, USA). BHB was quantified using the generated 8-point external calibration curve with Xcalibur Software 4.0 (Thermo Scientific Inc., Waltham, MA, USA).

### 2.6. Palmitate β-Oxidation

Palmitate β-oxidation in INS1E cells was measured in KRBH medium (0.2% BSA, 2 mM Ca^2+^, 1 mM Mg^2+^, 120 mM NaCl, 10 mM NaHCO_3_ 10 mM Hepes pH 7.4) supplemented with 2 mM glucose, 0.5 µCi radiolabeled [9, 10-3H]-palmitic acid (Perkin-Elmer, Waltham, MA, USA) and 250 µM unlabeled palmitic acid, as previously described [34]. The end products of fatty acid oxidation are H_2_O and CO_2_. At the end of the 2 h incubation time at 37 °C, the amount of ^3^H-labeled H_2_O that formed as a result of complete β-oxidation was measured after removal of the remaining palmitate using activated charcoal.

### 2.7. Mitochondrial Membrane Potential

The mitochondrial membrane potential (MMP) was measured in INS1E cells using the JC-10 Mitochondrial Membrane Potential Assay Kit (Abcam, Cambridge, UK) in accordance with the manufacturer’s recommendations. After treatment, cells were loaded with 1X JC-10 probe, dissolved in KRBH buffer for 45 min at 37 °C on a 96-welled plate with black walls. Using the citation 3 microplate reader with injectors, the excitation/emission (Ex/Em) of cells were monitored at 490/525 nm and 540/590 nm (cut off at 570 nm) through monochrometer filters. Changes in the MMP (Δψm) are expressed as the ratio of the fluorescence in mitochondria divided (525 nm) by the cytosolic fluorescence (590 nm) (Fmito/Fcyto), measured in the same cells over time, before and after glucose stimulation (16.7 mM). At the end of the recording, the protonophore, carbonyl cyanide-p-trifluoromethoxyphenylhydrazone (FCCP) was used to dissipate Δψm. Fluorescence ratios are reported as means + SD of a representative experiment in sextuplicates repeated three times.

### 2.8. Insulin Secretion Assay

INS1E cells, treated according to experimental conditions, were starved for 1 h in Krebs Ringer HEPES (KRBH) buffer in low glucose (2.8 mM) at 37 °C. The cells were then incubated in KRBH 2.8 mM glucose for 1 h (Basal) before stimulation with 16.7 mM of glucose for 1 h. Insulin released in the medium was determined by ELISA (ALPCO Diagnostics, Salem, NH, USA). Insulin concentration in nanograms per milliliter (ng/mL) was normalized against total protein in micrograms.

### 2.9. Animal Study

All animal care and procedures were approved by the Swiss Federal Food Safety and Veterinary Office (FSVO). Male Wistar rats, 21 months of age, were treated as previously described [16]. Rats were fed a standard chow diet (Provimi Kliba AG, Kaiseraugst, Switzerland), supplemented with 5% (*w*/*w*) medium chain triglycerides: octanoic triglycerides (MCT8), decanoic triglycerides (MCT10), MCT8:MCT10 (40:60%) or 5% sunflower oil (control) for 8 weeks. Sunflower oil was added to the standard chow diet in order to maintain an equivalent calorie and lipid content to that of the MCT-supplemented diets. At sacrifice, plasma was collected after at least 16 h fasting, and glycemia was measured using a glucometer. Pancreas’ from treated rats were isolated and prepared accordingly for experimental analysis.

### 2.10. Statistics

All experiments were performed in triplicate. Data are reported as means ± SEM. Data were analyzed with Student’s *t*-tests for paired observations. When comparing three or more means, ANOVAs were applied, followed by Dunnett’s multiple comparison tests. Values of * *p* < 0.05 and ** *p* < 0.01 were considered statistically significant.

## 3. Results

### 3.1. Medium Chain Fatty Acid Supplementation Improves β-Cell Function in Aged Rats

**Figure 1:**
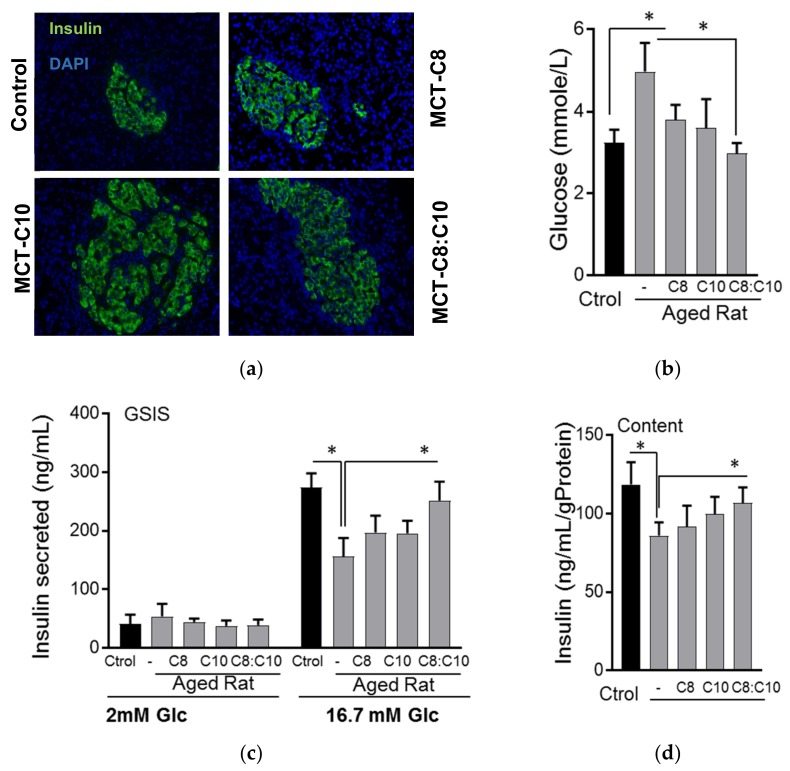
Medium chain fatty acids improved β-cell function in aged rats. (**a**) Immunohistochemical staining of pancreas with insulin from aged rats fed with normal chow diet supplemented with sunflower oil (Ctrol), octanoic acid (C8), decanoic acid (C10) or a 40:60 mixture of octanoic acid: decanoic acid (C8:C10) medium chain triglycerides for 8 weeks. (**b**) Fasting glucose (*n* = 12), (**c**) i secretion and (**d**) insulin content of young rats (3 months old) compared to aged rats (18 months old) treated as above after 16 h fasting (*n* = 6, * *p* < 0.05).

Considering the beneficial effects of a ketogenic diet on metabolic and brain health [35], we aimed to test whether an MCT-supplemented diet could improve metabolic health in aged rats. MCT-fed aged rats showed an increase in pancreatic islet area compared to sunflower oil-fed controls (Figure 1a). The rise in fasting glycemia observed in aged sunflower oil-fed control rats was significantly reduced in MCT-fed rats. Aged rats fed with the MCT C8:C10 mixture showed fasting glycemia comparable to healthy young rats (Figure 1b). To determine whether the MCT feeding affected β-cell function, we isolated primary islets from aged rats and performed glucose stimulated insulin secretion (GSIS). Our results showed a nearly 50% reduction in glucose stimulated insulin secretion in aged rats treated with sunflower oil, whereas the C8:C10 mixture rescued β-cell function to levels similar to the young control rats (Figure 1c) and significantly increased insulin content (Figure 1d).

### 3.2. C8 and C10 Differentially Activate FFAR1/GPR40 and Ketogenesis in β Cells

**Figure 2:**
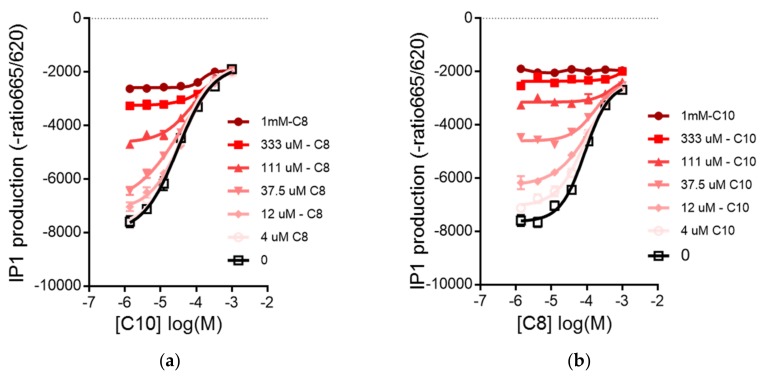

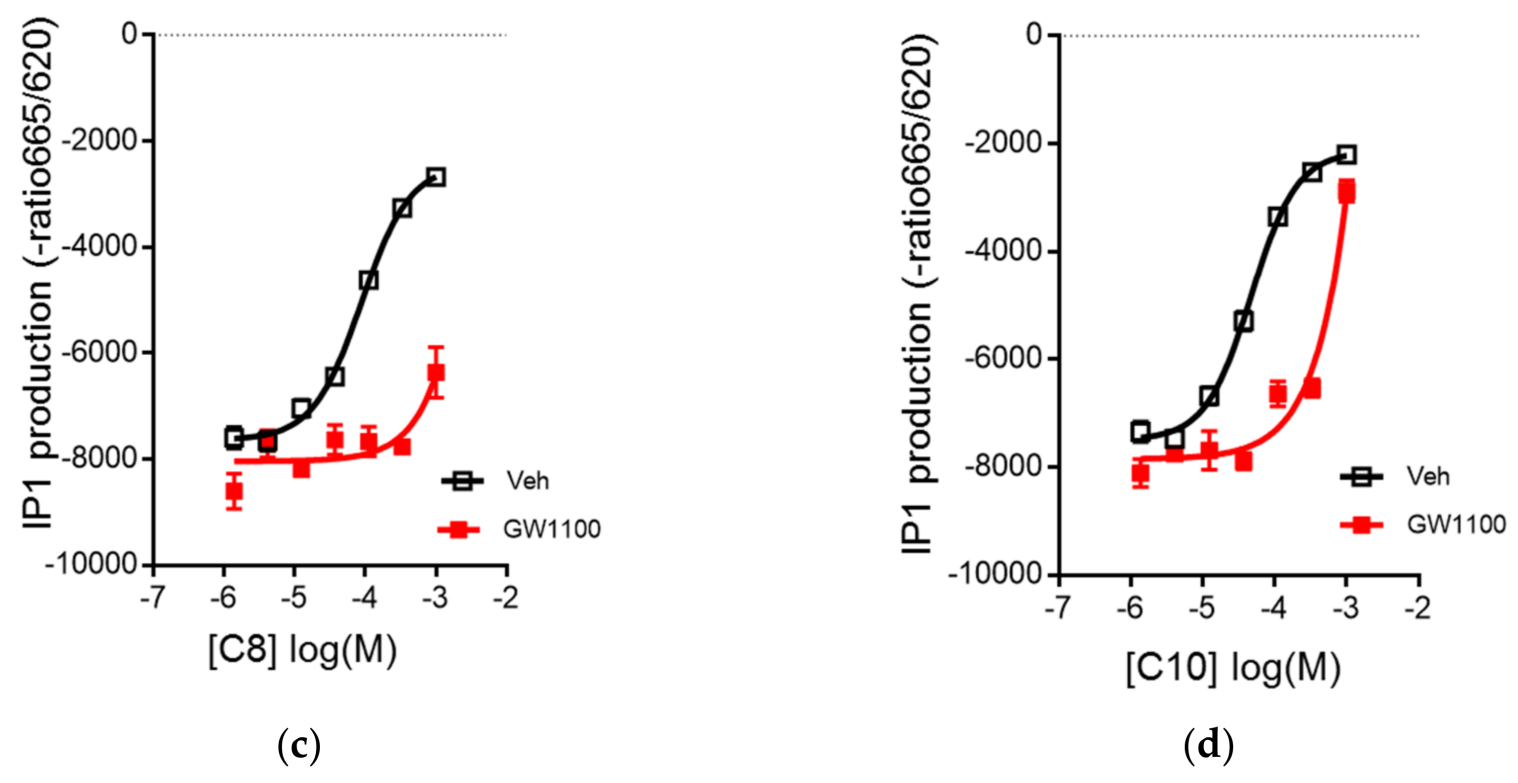
Activation of GPR40 by medium chain fatty acids, C8 and C10, in Chem1-GPR40 cells. (**a**) Dose–response curves of C8 in the presence of C10; dose–response of IP1 production with C8 activation in the presence of increasing concentrations of C10 and (**b**) dose–response curves of C10 in the presence of C8; C10 in the presence of increasing concentrations of C8. (**c**) Dose–response curves of C8 in GW1100; inhibitory effect of the GPR40 antagonist, GW1100, on the dose–response accumulation of IP1 by C8 or by (**d**) dose–response curves of C10 in GW1100; C10 in Chem1-GPR40 cells. Curves represent as means + SD of eight experimental replicates.

**Figure 3:**
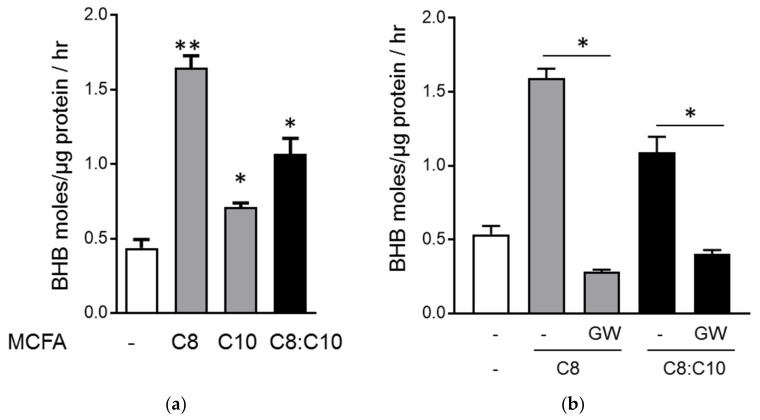

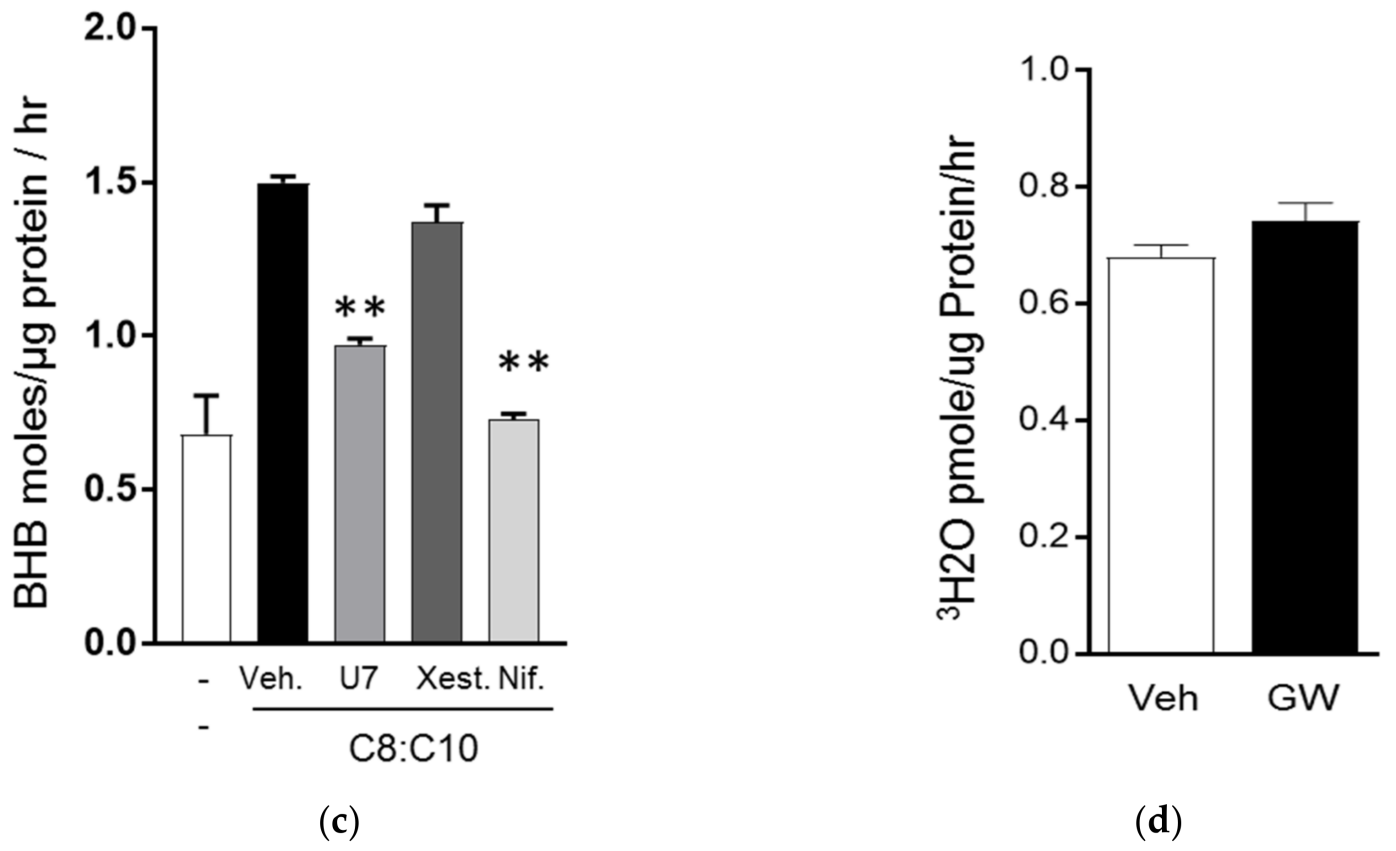
GPR40 signaling affects fatty acid β-oxidation in β-cells. (**a**) Ketone body β- hydroxybutyrate (BHB) production in INS1E β-cells in the presence of C8, C10 or C8:C10 40:60 mixture. (**b**) Inhibition of GPR40 signaling by the antagonist, GW1100 (GW), (**c**) the phospholipase C-β (PLCβ) inhibitor, U73122 (U7) and nifedipine (Nif) prevent BHB production from medium chain fatty acids (MCFA) in INS1E β-cells unlike the IP3 receptor inhibitor xantospongin (Xant). (**d**) Inhibition of GPR40 signaling by GW increased complete β-oxidation of palmitate. Data are presented as means + SEM of three independent experiments, * *p* < 0.05, ** *p* < 0.01 relative to control.

Aged rats fed with MCT-C8 and MCT-C10 were previously shown to have similar levels of circulating ketone bodies with high levels of circulating medium chain fatty acids (MCFA) C8 or C10 [16]. Under physiological conditions, the free fatty acids can activate FFAR1/GPR40 in β-cells to amplify GSIS. To assess the activation of the GPR40 receptor by MCFA, we measured the accumulation of inositol-1 phosphate (IP1), a degradation product of IP3, produced downstream of GPR40 signaling as a surrogate of receptor activation. In Chem-1 cells expressing GPR40, C8 (EC_50_: 143 µM) showed a lower affinity compared to C10 (EC_50_: 47.15 µM) (Figure 2a,b). We next evaluated whether the combination of C8 and C10 showed some synergy with IP1 accumulation after addition of each concentration by measuring the dose–response of C8 with increasing addition of C10 and vice versa. Our results showed that in both cases, the EC_50_ of C8 or C10 was not affected by the addition of the other MCFA, showing that C8 and C10 did not have any synergistic effect on IP1 accumulation in Chem-1 cells. However, in the presence of 37.5 μM of C8, the EC_50_ of C10 was marginally improved from 47.15 to 41.5 μM. In a competition assay using the GPR40 antagonist, GW1100 (50 µM), C8 activation of the receptor was totally abrogated, whereas C10 partially overcame the inhibition at the highest concentrations (Figure 2c,d). These results demonstrate that C8 and C10 show different affinities for GPR40 and might activate different molecular pathways in β-cells. It has been recently shown that the MCFA triggers a distinct effect in the energy metabolism of astrocytes with C8-stimulated ketogenesis, whereas C10 preferentially induces glycolysis [15]. Given these observations, we investigated the effects of MCFA C8, C10 or the C8:C10 mixture on mitochondrial function and ketogenesis in β-cells using C8 and C10 as substrates, either alone or in combination. In β-cells, C8 is a much better substrate for ketogenesis compared to C10, and the C8:C10 mixture showed an intermediate effect (Figure 3a). We next determined whether the observed effect of MCFA on mitochondrial ketogenesis was affected by GPR40 signaling. Inhibition of GPR40 activation by the antagonist, GW1100, completely abrogated ketogenesis from C8 and the C8:C10 mix in INS1E cells (Figure 3b). Furthermore, inhibition of signaling cascades downstream of GPR40 activation, such as the l-type Ca^2+^ channel by nifedipine or PLCβ by U73122, significantly repressed BHB production, whereas inhibition of the IP3 receptor with Xestospongin C showed no effect (Figure 3c). However, inhibition of GPR40 signaling by GW1100 increased complete palmitate β-oxidation, measured by generation of radiolabeled H_2_O (Figure 3d), suggesting that GPR40 signaling promotes ketogenesis with minimal effects on ATP production in β-cells (Krebs cycle and oxidative phosphorylation). It is likely that PKC activation by DAG and possibly calcium influx downstream of GPR40 activation contribute to the ketogenesis measured by BHB production in β-cells.

### 3.3. MCFA Acutely Increased Insulin Secretion with Beneficial Long-Term Effects

**Figure 4:**
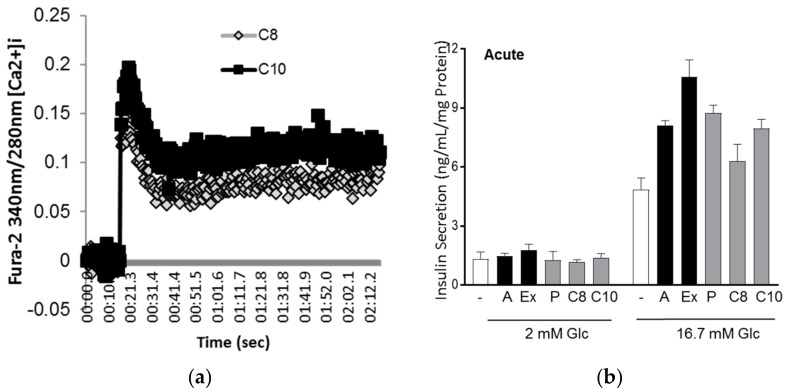

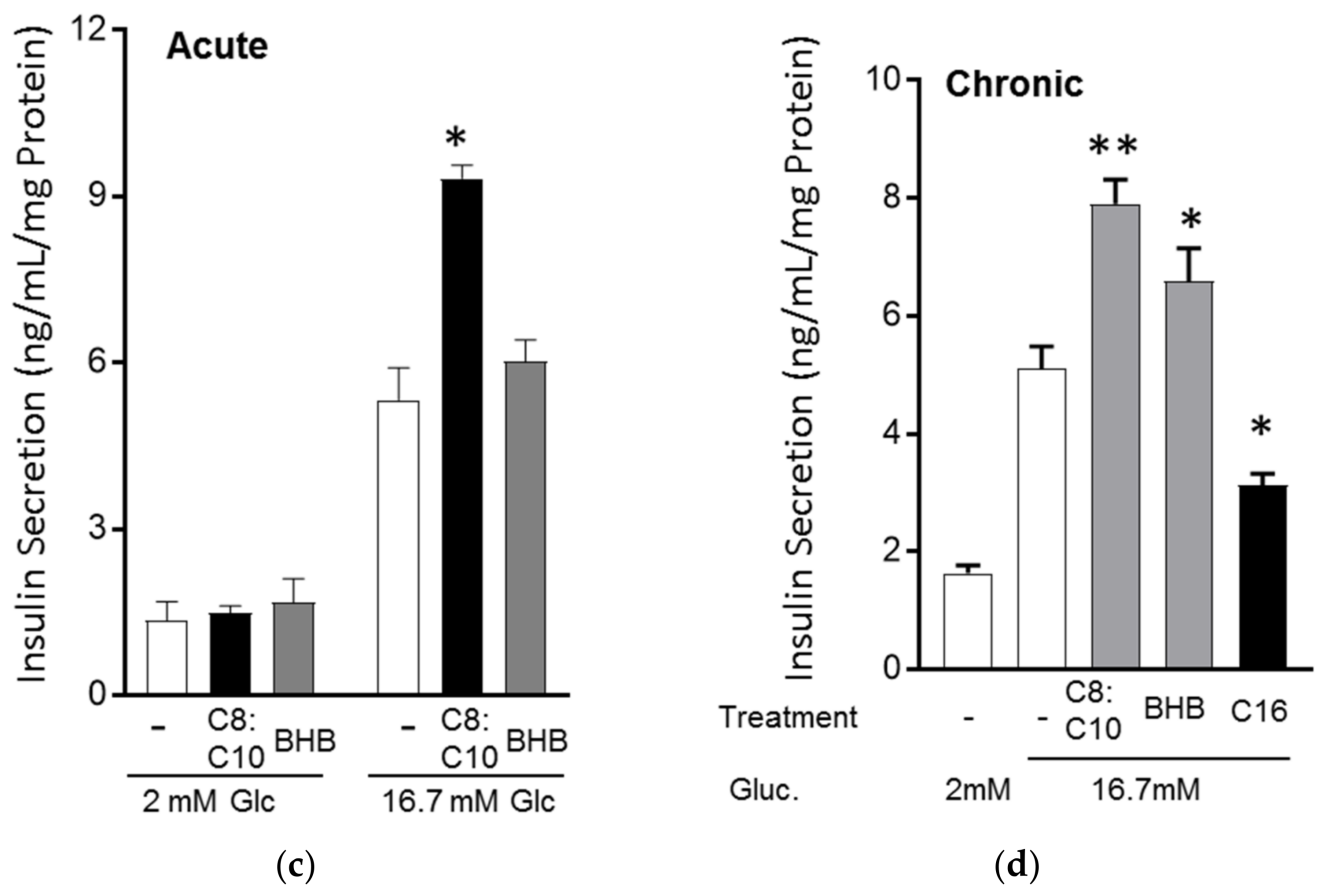
Regulation of insulin secretion by MCFA and BHB in INS1E cells. (**a**) Increase of calcium mobilization by C8 and C10. (**b**) Acute statistic insulin secretion after 1 h of treatment with secretagogues, like 1× amino-acids (A), 0.1 µM Exendin-4 (Ex) and 200 µM fatty acids—palmitate (PA, C16:0), C8, C10, in low (2 mM) and high (16.7 mM) glucose or (**c**) 200 µM C8:C10 40:60 mixture or 200 µM of BHB. (**d**) Static insulin secretion after chronic (72 h) treatment with 200 µM of C8:C10 40:60 mixture, BHB or palmitate (P). Data represent means + SEM of three independent experiments, * *p* < 0.05, ** *p* < 0.01 relative to control.

Activation of GPR40 amplified glucose induced insulin secretion, in part by increasing calcium influx from the l-type calcium channel [29]. Indeed, as a better GPR40 agonist, C10 showed a higher activation of calcium mobilization in INS1E cells compared to C8 (Figure 4a). Unlike C8, addition of C10 (Figure 4b) and the C8:C10 mix (Figure 4c) potentiated GSIS to the same extent as classical secretagogues, such as amino-acids; the Glp1r agonist, Exendin-4; or long chain fatty acids like palmitate (Figure 4b). As previously reported [36], BHB had no direct effect on GSIS (Figure 4c), whereas cells chronically treated with the 200 µM C8:C10 mix or with BHB showed a significant improvement in GSIS. On the other hand, chronic palmitate treatment induced marked β-cell dysfunction (Figure 4d) resulting from ER and oxidative stress [37]. Unlike saturated long chain fatty acids, MCFA C8:C10 offers beneficial effects to chronically treated β-cells in part through the generated ketone body, BHB.

### 3.4. MCFA Help β-Cells Recover from Dysfunction

**Figure 5:**
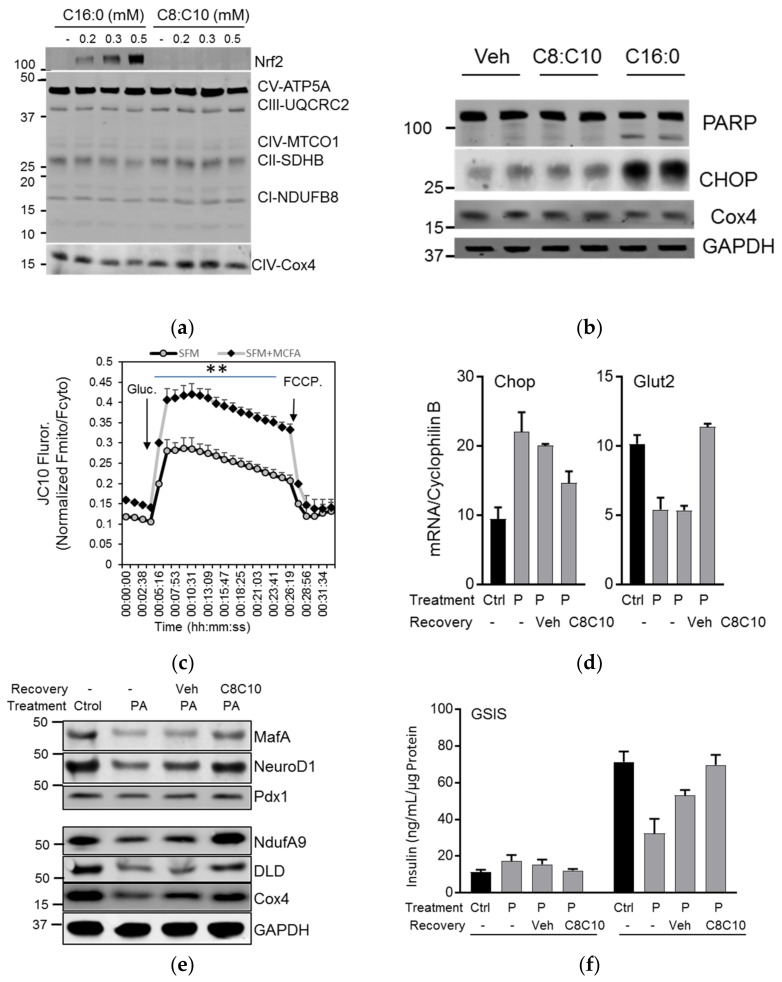
MCFA helps the β-cells recover from chronic palmitate induced dysfunction. (**a**) Representative immunoblotting of INS1E cells after chronic treatment with increased concentrations of palmitate or MCFA C8:C10 mixture with antibodies against Nrf2 and mitochondrial respiratory chain proteins. (**b**) Assessment of apoptotic and ER stress signaling after chronic treatment with palmitate (C16:0) or MCFA C8:C10 mixture after immunoblotting with antibodies against PARP (Poly(ADP-Ribose) Polymerase), CHOP, Cox4 (Cytochrome C Oxidase Subunit 4) and GAPDH (Glyceraldehyde-3-Phosphate Dehydrogenase) as loading controls. (**c**) Mitochondrial membrane potential (MMP) in response to 16.7 mM glucose in INS-1E cells chronically treated with vehicle control (Ctrl) or the MCFA C8:C10 mixture, using the fluorescent lipophilic cation IP10. Changes in MMP are expressed as the ratio of the fluorescence in mitochondria divided by the cytosolic fluorescence (Fmito/Fcyto) measured in the same cells. At the end of the recording, the protonophore, FCCP (2 μM) was used to dissipate MMP. Data shown are the means ± SEM from three independent experiments done in sextuplet in 96-welled plates, ** *p* < 0.01. (**d**) Expression of CHOP and Glut2 by qPCR in INS1E cells after chronic treatment with palmitate and recovery in control medium or MCFA-enriched medium. (**e**) Western blot analysis of transcription factors regulating β-cell function (MafA, NeuroD1, PDX1) or mitochondrial proteins (NDUFA9, DLD, Cox4) after treatment as above; GAPDH was used as a loading control. (**f**) Analysis of β-cell function after impairment with chronic palmitate and recovery as above by insulin secretion under basal (2 mM glucose) and stimulated (16.7 mM glucose + 0.1 µM Ex-4) conditions. Data represent means + SEM of three independent experiments; * *p* < 0.05, ** *p* < 0.01 relative to control cells and # *p* < 0.05 relative to palmitate control cells.

Elderly and obese adults are at high risk for the development of type 2 diabetes as a result of increasing insulin resistance and impaired pancreatic β-cell function. The elevation of circulating free fatty acids is a common feature observed in these individuals. In β-cells chronically treated with fatty acids, palmitate induced ER stress by increasing the expression of Nrf2, reducing the expression of mitochondrial respiratory chain proteins in a concentration dependent manner (Figure 5a) and triggered apoptosis and ER stress (Figure 5b), while C8:C10 treatment induced no adverse effects. Although MCFA C8 and C10 showed a small effect on the mitochondrial oxygen consumption rate (OCR) in INS1E cells, the MCFA mixture substantially increased OCR (Figure S1). Moreover, chronic treatment with MCFA significantly increased the glucose induced mitochondrial membrane potential (Figure 5c). These findings suggest that the beneficial effect of MCFA C8:C10 on β-cell function might stem from improved mitochondrial function. To test whether MCFA increase β-cell recovery and protection from chronic lipotoxicity, we cultured INS1E cells in the presence of 400 µM of palmitate in RPMI supplemented with 5% FBS for 72 h to induce β-cell dysfunction through lipotoxicity. The dysfunctional β-cells were left to recover for 48 h in complete medium with 5% FBS, or they were supplemented with C8:C10 (Figure S2A). Chronic palmitate treatment induced dysfunction, exemplified by increased expression of the ER stress activated gene C/EBP homologous protein (CHOP) and reduced Glut2 expression (glucose sensing), while medium supplemented with C8:C10 mix (100 µM) significantly restored the expression of these genes to control levels (Figure 5d). The expression of transcription factors involved in insulin biosynthesis and β function [38] that were reduced after palmitate treatment, like MafA and NeuroD1, were restored in the presence of the C8:C10 mixture (Figure 5e, Figure S2B). During recovery from lipotoxicity induced by palmitate, the C8:C10 MCFA mix showed similar beneficial effects on the expression of key metabolic genes involved in oxidative phosphorylation, such as NdufA9 (Complex 1), and CoxIV (Complex 4) as well as genes involved in energy metabolism like Dihydrolipoamide Dehydrogenase (DLD) (Figure 5e, Figure S2B). Pancreatic β-cells chronically treated with palmitate were dysfunctional with a 60% reduction in GSIS; recovery in complete medium partially restored β-cell function, but the presence of the C8:C10 mix restored function to control levels after 48 h (Figure 5f).

In summary, the medium chain fatty acid C8:C10 mixture improved mitochondrial function and restored GSIS defects after lipotoxic stress induced by chronic palmitate treatment, by increasing the expression of key functional genes involved in β maturation and function.

**Figure 6:**
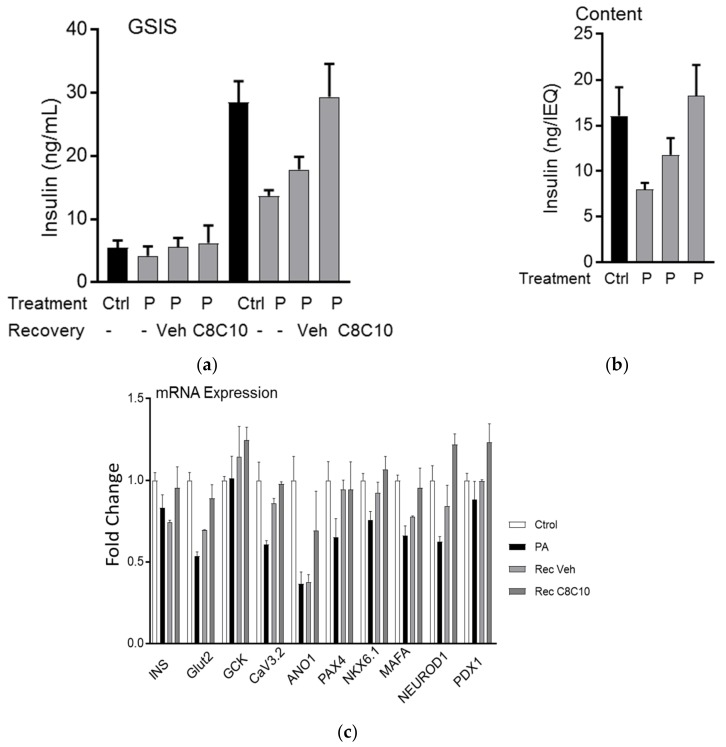
Chronic MCFA treatment improves β-cell function in human islets. (**a**) Glucose stimulated insulin secretion (GSIS) and (**b**) insulin content in healthy human islets treated with palmitate (P, 0.5 mM for 72 h) followed by recovery in the presence or absence of the medium chain fatty acid mixture, C8:C10 40:60. (**c**) Fold change of selected genes in human islets treated as above, using a NanoString n-counter gene expression analysis.

**Figure 7:**
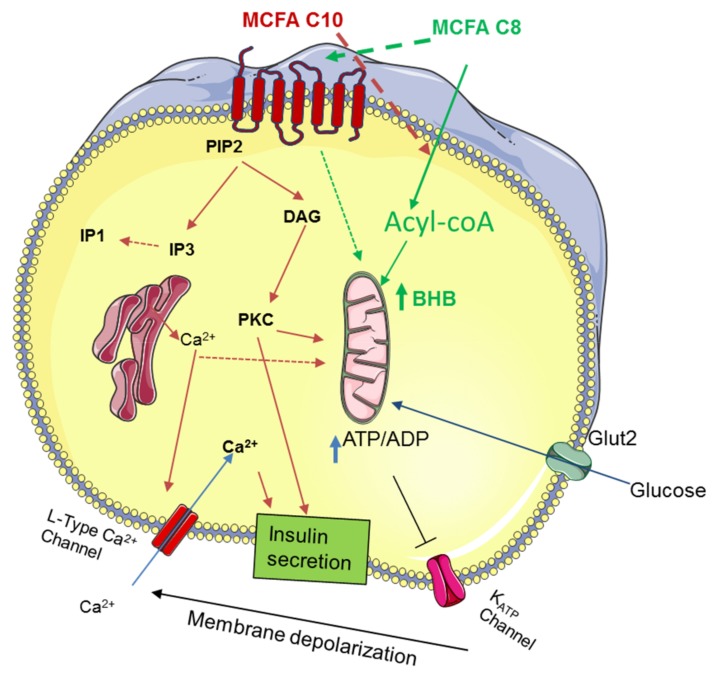
Model of MCFA signaling in β-cells. Pancreatic β-cell function was assessed by the capacity of the β-cell to sense and respond to glucose stimulation through uptake by Glut2 and mitochondrial metabolism leading to an increased ATP/ADP. Inhibition of the kATP channel by ATP leads to membrane depolarization and influx of calcium through the l-type calcium channel that triggers insulin secretion. Preferentially, the activation of GPR40 by C10 MCFA leads to PLCβ activation and generation of IP3 induced ER-calcium release and DAG activation of PKC, which amplifies glucose stimulated insulin release. C8, on the other hand, preferentially increases BHB production. GPR40 activation promotes mitochondrial ketogenesis through Ca^2+^ and certainly through DAG-PKC signaling. The illustration was made by using tools from Servier Medical Art, http://www.servier.fr/ servier-medical-art.

We next investigated the relevance of MCFA in human islets chronically treated with 500 µM of palmitate for 72 h. The C8:C10 mixture completely restored the defects in β cell function (Figure 6a) and insulin content (Figure 6b) observed in human islets chronically treated with palmitate. Using NanoString n-counter for gene expression profiling, we assessed gene expression changes in human islets to confirm our observations in INS1E cells. Our results showed that in human islets, the C8:C10 mix rescued the expression of genes negatively affected by palmitate, such as MafA, NeuroD1, Pax4, involved in insulin biosynthesis, glucose transporter 2 (Glut2) and channels regulating insulin secretion (ANO1, Cav3.2) but did not affect Pdx1 and glucokinase (Gck) expression levels (Figure 6c). Collectively our findings prove that MCFA protects human and rodent β-cells from lipotoxic dysfunction impinging on gene expression and mitochondrial function (Figure 7). 

## 4. Discussion

Increased β-cell dysfunction, exacerbated by reduced islet neogenesis and β-cell proliferation in human 35, contributes to the prevalence of type 2 diabetes in the elderly population. It has been noted that in some cases of type 2 diabetes and prediabetes, lifestyle interventions such as caloric restriction could be sufficient to improve β-cell function and glucose tolerance [39]. Ketogenic diets have been developed to mimic some of the health benefits of caloric restriction, and the therapeutic potential of associated ketogenesis in pathological conditions such as diabetes and neurological diseases is well documented [17,40]. However, the direct effect of the ketogenic diet on β-cell function is poorly understood. In this study, we demonstrated that aged rats fed a standard diet supplemented with 5% medium chain triglycerides, MCT C8 and MCT C10, exhibited improved glycemia compared to control animals fed with an isocaloric diet supplemented with sunflower oil (Figure 1). In this context, the MCT C8:C10 40:60 mixture improved β-cell function and insulin content to levels similar to young control rats. In several studies, C8 and C10 have shown differential metabolic effects [15,16,41]; however, their combined effect in metabolic health has not been clearly assessed. In addition to being an essential energy source, fatty acids function as signaling molecules that regulate various cellular processes based on their carbon length and degree of saturation. Here, we show that C10 is a better activator of GPR40 compared to C8 (Figure 2), but C8 is a better ketogenic substrate (Figure 3) and the combination of C8 and C10 can effectively activate GPR40 while increasing BHB production to a significant level (Figure 3). Here, we show that GPR40 activation is necessary for ketone body production in β-cells (Figure 3C), and pharmacological inhibition of the receptor led to reduced ketogenesis and increased complete fatty acid β-oxidation (Figure 3D). The cross talk between GPR40 signaling and mitochondrial activity can arise from ER Ca^2+^signaling through IP3 receptor activation and/or DAG-activated PKC39. Although inhibition of upstream pathways such as the l-type Ca^2+^ channels by nifedipine or the PLCβ activity significantly reduced BHB production, the IP3 receptor showed minimal effect. It is likely that GPR40 activation by the MCFA C8:C10 mix enhanced mitochondrial function and BHB production in pancreatic β-cells.

Although BHB has no acute effect on insulin secretion (Figure 4b,c), chronic treatment of INS1E cells with the C8:C10 mix or BHB alone significantly improved GSIS (Figure 4d) contrary to the effect of saturated LCFAs like palmitate. Ketone bodies have beneficial effects related to improving metabolic outcomes in type 2 diabetes and normalization of β-cell function [39,42]. Excessive saturated LCFAs such as palmitate have detrimental effects and induce lipotoxicity, ER stress, apoptosis and β-cell dysfunction, while MCFA shows no long-term cytotoxic effects in β-cells (Figure 5). We examined the effect of MCFA in the functional recovery of β cells using a model of palmitate induced β-cell dysfunction [43]. Pancreatic β cells can completely recover from lipotoxicity-induced impaired GSIS after four days of recovery in complete medium without palmitate [44]. In our model, INS1E cells chronically treated with a low concentration of palmitate (300 µM for 72 h) underwent a reversible lipotoxic response with limited effects on cell survival. Addition of MCFA in the recovery medium restored GSIS to levels comparable to control cells after just 48 h in INS1E (Figure 5e) and human islets (Figure 6a) while increasing the insulin content. MCFA treatment improved ER stress with reduced CHOP expression (Figure 5a), and the functional recovery of GSIS may have resulted from increased expression of Glut2, mitochondrial respiratory complex proteins (Cox4, NdufA9) and DLD, a relevant part of the pyruvate dehydrogenase complex involved in mitochondrial energy metabolism 43. Pancreatic β-cell recovery with MCFA also increased genes involved in insulin biosynthesis pathways (MafA, NeuroD1) observed in both INS1E (Figure 5) and human islets (Figure 6). Age-related β-cell dysfunction can be attributed to mitochondrial dysfunction, reduced expression of Glut2 or accumulation of advanced glycation end products (AGEs) [45], and the beneficial effect of MCT in β-cells might be relevant for improving metabolic health during aging Several studies have previously investigated the relationship between ketogenesis, in particular regarding BHB, and β cell function, albeit with conflicting results. Zhou et al. showed that chronic exposure of high concentrations of BHB (up to 10 mM) reduced GSIS44, whereas others demonstrated the beneficial effect of BHB in β-cell function [36,46]. These inconsistent data might be a result of the concentrations of BHB or the nature of the fatty acids used. Here, we showed that the MCFA C8:C10 mixture was able to concomitantly stimulate GPR40 and mitochondrial ketogenesis as well as induce low concentrations BHB (0.2 mM); this could be due to its effects against inflammation 46 and oxidative stress [47].

## 5. Conclusions

This study compared the functional roles of C8 and C10 and their combined effect on pancreatic β-cell function under various pathological conditions. Our findings confirmed previous reports suggesting the beneficial effect of ketogenic diet on β-cell function. The C8:C10 mixture induced ketogenesis while activating the GPR40 receptor and restored β-cell function in aged rats and sped up the functional recovery from lipotoxicity in human and rat β-cell lines. MCFA and ketones showed benefits following long-term treatment and may function as regulatory metabolites that affect gene expression and mitochondrial function. In the elderly, the possibility that dietary interventions like an MCFA-based ketogenic diet could be useful alternatives to promote the reduction of pharmacological methods of treatment, which are often lifelong with significant side effects.

## Supplementary Materials

The following are available online at http://www.mdpi.com/2072-6643/10/4/473/s1, Figure S1: Oxygen consumption rate (OCR) measured in INS1E cells after stimulation with 2 mM glucose (control CTRL), 20 mM glucose or 20 mM glucose with the 100 μM of MCFA C8, C10 or C8:C10 40:60 mix; Figure S2: Protein expression changes during recovery from lipotoxicity induced by PA.
