# Supplementary material for: Coordination of GPR40 and Ketogenesis Signaling by Medium Chain Fatty Acids Regulates Beta Cell Function
Source: Nutrients. 2018 Apr 12;10(4):473. doi: 10.3390/nu10040473 (PMC5946258; doi:10.3390/nu10040473)
Supplement: Supplementary file 1 [file nutrients-10-00473-s001.pdf]

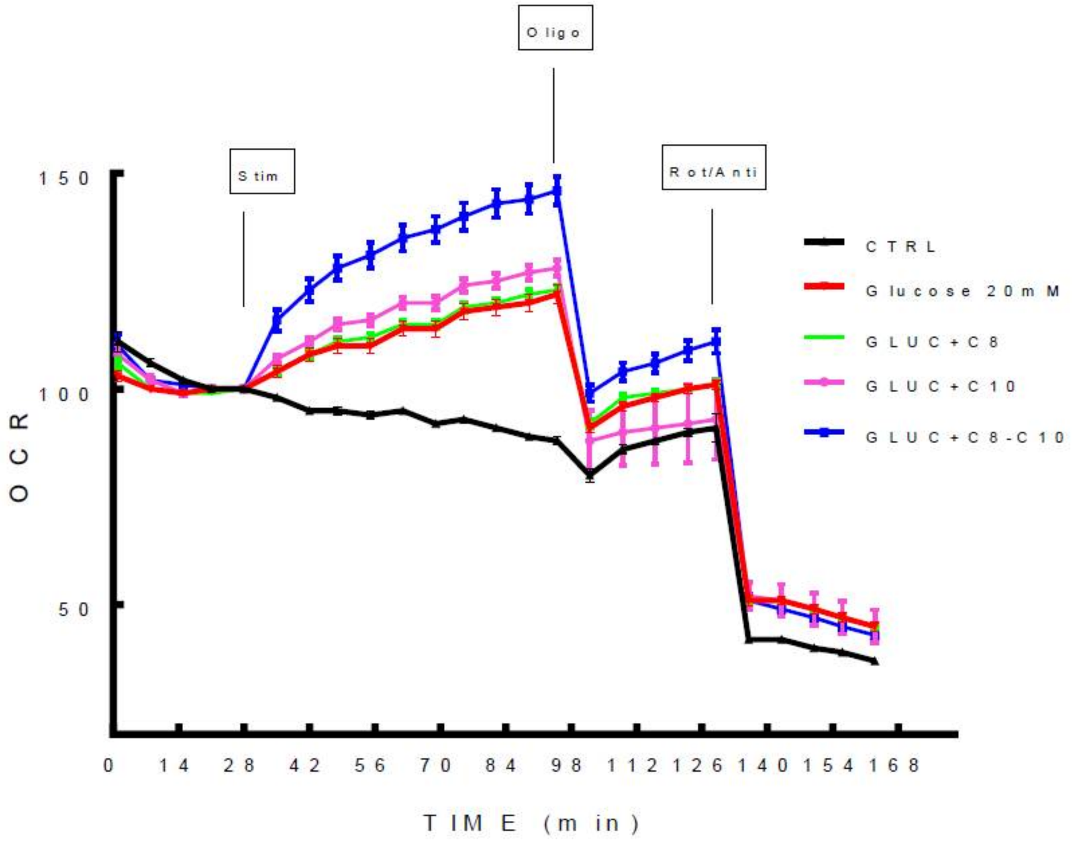

Figure S1. Oxygen consumption rate (OCR) measured in INS1E cells after stimulation with 2 mM glucose (control CTRL), 20 mM glucose or 20 mM glucose with the 100 µM of MCFA C8, C10 or C8:C10 40:60 mix.

OCR is a well-established protocol using the with Seahorse XF-96 extracellular flux analyzer to measure mitochondrial function. After starvation in KRBH 2 mM glucose for 2 h to measure basal respiration, cells were stimulated as described, followed by inhibition of ATP synthase by oligomycin (ATP dependent respiration) and mitochondrial respiration by rotenone and antimycin A (Rot/Anti) to shut down total respiration.

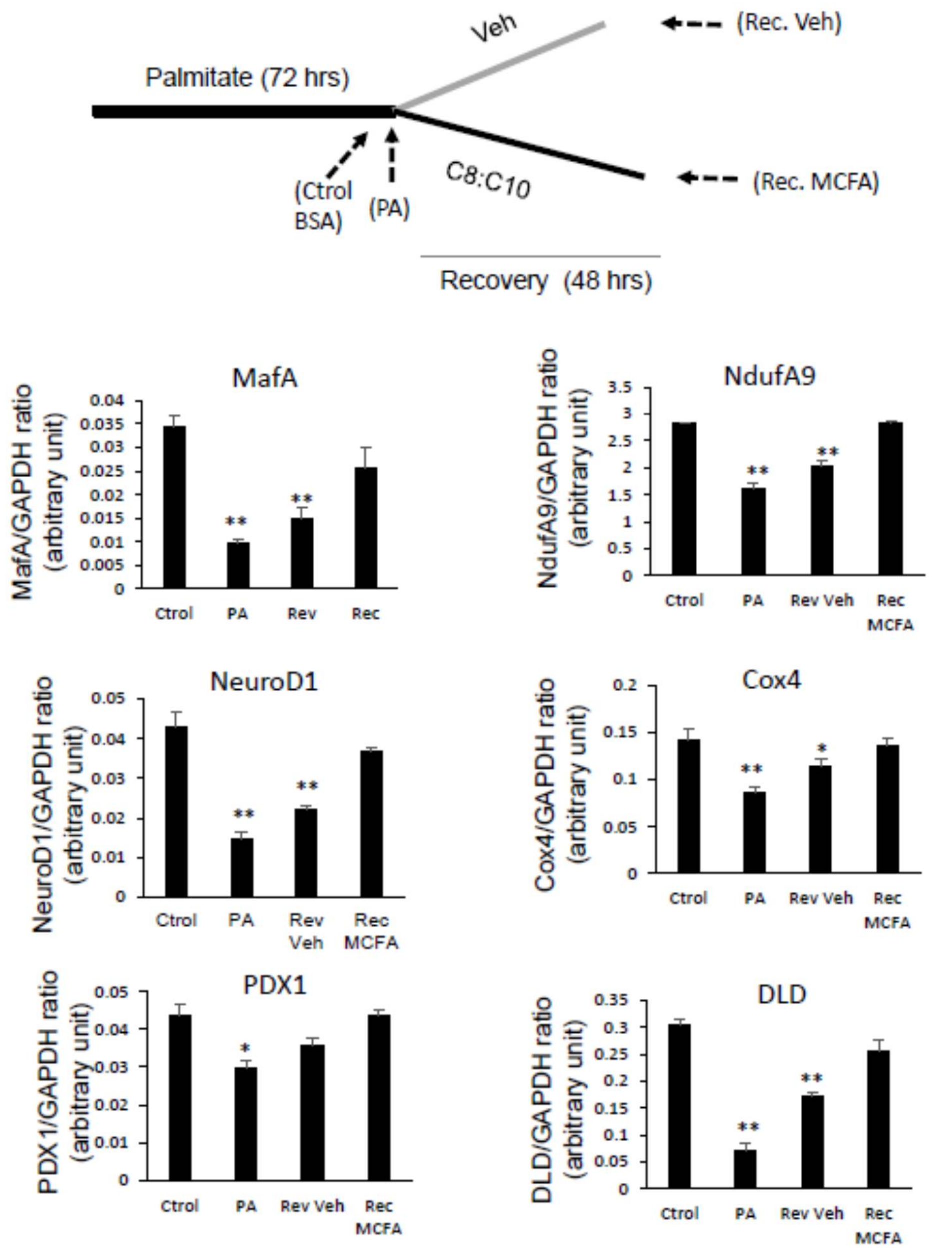

Figure S2. Protein expression changes during recovery from lipotoxicity induced by PA.

(A) Design of the β cell recovery experiment. INS1E cells treated with BSA were used a controls (Ctrol). After 72 h treatment with 400 μM palmitate (PA) before recovery in RPMI medium containing 100 μM of the C8:C10 40:60 ratio (MCFA) or vehicle (veh). (B) The relative protein expression was determined by the ratio of the sample value relative to the internal loading control GAPDH using the Licor Odyssey and the Image Studio analysis software. Values are mean + SD, n=3. **p<0.05, **p<0.01 relative to control.
